# *S*-Nitrosoglutathione Reductase Plays Opposite Roles in SH-SY5Y Models of Parkinson's Disease and Amyotrophic Lateral Sclerosis

**DOI:** 10.1155/2015/536238

**Published:** 2015-09-27

**Authors:** Salvatore Rizza, Claudia Cirotti, Costanza Montagna, Simone Cardaci, Claudia Consales, Mauro Cozzolino, Maria Teresa Carrì, Francesco Cecconi, Giuseppe Filomeni

**Affiliations:** ^1^Cell Stress and Survival Unit, Danish Cancer Society Research Center, 2100 Copenhagen, Denmark; ^2^IRCCS Santa Lucia Foundation and Department of Biology, University of Rome “Tor Vergata”, 00133 Rome, Italy; ^3^The Beatson Institute for Cancer Research, Cancer Research UK, Glasgow G61 1BD, UK; ^4^Unit of Radiation Biology and Human Health, ENEA-Casaccia, 00123 Rome, Italy; ^5^Institute for Translational Pharmacology, CNR, 00133 Rome, Italy

## Abstract

Oxidative and nitrosative stresses have been reported as detrimental phenomena concurring to the onset of several neurodegenerative diseases. Here we reported that the ectopic modulation of the denitrosylating enzyme *S*-nitrosoglutathione reductase (GSNOR) differently impinges on the phenotype of two SH-SY5Y-based *in vitro* models of neurodegeneration, namely, Parkinson's disease (PD) and familial amyotrophic lateral sclerosis (fALS). In particular, we provide evidence that GSNOR-knocking down protects SH-SY5Y against PD toxins, while, by contrast, its upregulation is required for G93A-SOD1 expressing cells resistance to NO-releasing drugs. Although completely opposite, both conditions are characterized by Nrf2 localization in the nuclear compartment: in the first case induced by GSNOR silencing, while in the second one underlying the antinitrosative response. Overall, our results demonstrate that GSNOR expression has different effect on neuronal viability in dependence on the stimulus applied and suggest that GSNOR could be a responsive gene downstream of Nrf2 activation.

## 1. Introduction

Oxidative and nitrosative stress has been copiously reported to play a pivotal role in the onset of neurodegenerative diseases [1, 2]. Both endogenous and exogenous sources of nitrooxidative stress, such as mitochondria [3] and immune cells [4] that produce reactive oxygen species (ROS) or reactive nitrogen species (RNS) deriving from nitric oxide (NO), deeply contribute to the persistence of noxious conditions affecting neuron viability. Parkinson's disease (PD) and amyotrophic lateral sclerosis (ALS) are among the neurodegenerative disorders in which nitrooxidative stress has been particularly reported being implied in the loss of specific neuronal populations.

PD is a neurodegenerative disorder that leads to a progressive loss of dopaminergic neurons. The mechanisms underlying this phenomenon are not completely clarified. However, mitochondrial damages (mainly those affecting complex I of the respiratory chain), failure in proteins, and organelles quality control as well as oxidative and nitrosative stress have been suggested to be implicated in the pathogenesis of PD [5]. Several are the mutations associated with the family forms of PD [6]. Many of them affect specific proteins, for example, PINK1, Parkin, and DJ-1. In particular, DJ-1 has been implicated in the protection against oxidative insults as it interferes with the production of mitochondrial ROS, and it has been reported to be undergoing activation by* S*-nitrosylation processes [7–9]. Moreover, the existence of a signaling axis involving DJ-1 as upstream modulator of Nrf2 (NE-F2-related factor-2) [10, 11], the transcription factor known as one of the master regulators of redox homeostasis, has recently been demonstrated, suggesting that DJ-1 may act in different ways in order to counteract oxidative stress.

ALS is a multifactorial disease caused by motor neuron degeneration that can be divided into a sporadic (90% of the cases) and a familial form (fALS) whose 20% of mutations (2% of total ALS cases) map throughout the* SOD1* gene (coding the Cu, Zn superoxide dismutase) [12, 13]. More than 100 point mutations in SOD1 gene are associated with fALS cases, with some of them affecting its antioxidant activity. One of the most studied SOD1 mutations is the G93A substitution which results in the aggregation of the mutant forms, being, in such a way, detrimental for mitochondrial homeostasis and motor neuron survival. Another common feature of ALS is the chronic inflammatory response activated in the cells surrounding motor neurons (astrocytes and microglia) that leads to the production of cytokines and NO [14–16].

*S*-Nitrosylation represents one of the main NO-mediated posttranslational modifications [7]. It is based on the attachment of an NO moiety to the thiol group (-SH) of cysteine residues, resulting in the formation of low (amino acids and peptides) and high molecular weight (proteins)* S*-nitrosothiols (SNOs). Although NO could modulate either neuronal viability or death, the role of neuronal* S*-nitrosothiols (SNOs) in the etiopathogenesis of neurodegenerative diseases remains to be fully elucidated yet. Protein aggregates found in brains from PD and ALS patients show a great abundance of nitrated/*S*-nitrosylated proteins, suggesting that SNOs are involved in the pathogenesis and/or in the neuronal defense responses. However, recent results [17] indicate that NO is a potent antioxidant agent that reduces oxidative stress in the central nervous system, such as by increasing the antioxidant capacity of reduced glutathione (GSH) through the formation of* S-*nitrosoglutathione (GSNO), which is a 100-fold more potent antioxidant with respect to GSH.

It has been reported that class III alcohol dehydrogenase [18] also known as* S*-nitrosoglutathione reductase (GSNOR) catalyzes the degradation of GSNO, thus regulating the amount of SNOs and NO bioactivity. Although GSNOR-deficient (KO) mice are viable and fertile, they show some pathological phenotypes, many of which related to aging, such as (i) compromised lymphocytes development [19]; (ii) high morbidity and mortality upon endotoxic shock [20]; (iii) DNA damage accumulation; (iv) high probability to develop hepatocellular carcinoma [21, 22]; (v) early signs of peripheral neuropathy [23]. Altogether, this evidence indicates that (i) excessive* S*-nitrosylation negatively impacts on cell physiology and (ii) GSNOR acts as the principal molecular modulator underneath. GSNOR, in fact, is the best characterized denitrosylating enzyme evolutionarily conserved from bacteria to humans [24], which is widely expressed in all tissues and organs. In particular, brain has been reported to express high levels of GSNOR [24]. However, any alteration linked to GSNOR deficiency in nervous system has never been reported so far.

Based on the evidence that GSNOR deficiency results in ageing-related phenotypes and that brain represents the organ mostly affected during ageing, here we have aimed at characterizing the role of GSNOR in modulating the severity of two neurodegenerative diseases, PD and fALS, taking SH-SY5Y as elective experimental system.

## 2. Materials and Methods

### 2.1. Cell Cultures

Human SH-SY5Y cells were purchased from the European Collection of Cell Culture and grown at 37°C in an atmosphere of 5% CO_2_. SH-SY5Y cells were cultured in DMEM-F12 containing 25 mM glucose, supplemented with 10% fetal bovine serum, 2 mM L-glutamine, and 1,000 U/mL penicillin-streptomycin. All media were from Lonza. G93A-SOD1 stably expressing SH-SY5Y cells were generated as previously reported [25].* LowNO* clone was selected from a mixed population of G93A cells subjected to repeated treatments with GSNO.

### 2.2. Cell Transfection with siRNAs

24 hours after plating, SH-SY5Y cells were transfected with a commercially available small interference RNA (siRNA) against GSNOR (Sigma). Control cells were transfected with a scramble siRNA duplex (siScr), which does not present homology with any other human mRNAs. In experiments aimed at downregulating DJ-1, double transfection (with both siRNAs against GSNOR and DJ-1) was carried out simultaneously.

### 2.3. Treatments

All treatments were performed for 24 h in completed culture media. DPTA was used at a concentration of 400 *μ*M, H_2_O_2_ at 1 mM, MPP^+^ at 1.25 or 2.5 mM, and 6OHDA at 50 *μ*M. Trigonelline was preincubated for 30 min before treatments with DPTA and used at a concentration of 0.5 or 1 *μ*M.

### 2.4. Analysis of Cell Death

Cell death was evaluated by direct cell count at optic microscopy upon Trypan blue staining or, cytofluorometrically, by assessing the percentages of apoptotic cells upon staining with propidium iodide.

### 2.5. Animal Experimentation

All mouse experiments were conducted in accordance with the European Community guidelines and with the approval of relevant national (Ministry of Health) and local (Institutional Animal Care and Use, Tor Vergata University) ethical committees. All mice employed in the experiments (wild type, G93A, and GSNOR-KO) were on C57BL/6J background. They have been raised and crossed in the indoor animal house in a 12 h light/dark cycle in a virus/antigen-free facility with controlled temperature and humidity and have been provided with water and food* ad libitum*. At the indicated time, mice were anesthetized with ketamine–xylazine, killed, and dissected for the different experiments. All efforts were made to minimize suffering.

#### 2.5.1. Spinal Cords

Spinal cords were collected from wild type (WT) and symptomatic (135-day-old) G93A-SOD1 mice B6.Cg-Tg(SOD1-G93A)1Gur/J (Jackson Laboratory, Bar Harbor, ME, USA) minced and lysed by 40–60 strokes in Potter-Elvehjem homogenizer and supernatants used for PSNOs detection and GSNOR activity.

#### 2.5.2. Brains

Brains were collected from 2-month-old wild type (WT) and GSNOR-deficient (KO) mice. Mesencephalon (ventromedial zone) was then isolated, since it contains most of the dopaminergic cells (which are affected in PD), minced and lysed by 30–40 strokes in Potter-Elvehjem homogenizer and supernatants used for Western blot analyses.

#### 2.5.3. PCN

Mouse primary cortical neurons (PCN) were obtained from cerebral cortices of E15 C57BL/6 mice (WT and GSNOR-KO) embryos. Minced cortices were digested with trypsin EDTA 0.25% at 37°C for 7 minutes. Cells were stained with 0.08% Trypan blue solution and only viable cells were counted and plated at the density of 1 × 10^5^/cm^2^ onto poly-d-lysine coated multiwell plates in 25 mM glucose-containing MEM medium supplemented with 10% fetal bovine serum, 2 mM glutamine, and 0.1 mg/mL gentamicin. After 1 hour, the medium was replaced with Neurobasal medium containing antioxidant-free B27 supplement, 2 mM glutamine, and 0.1 mg/mL gentamicin. Cell cultures were kept at 37°C in a humidified atmosphere containing 5% CO_2_. Every 3 days, one-third of the medium was replaced up to day 7, the time at which the cells were treated.

### 2.6. Western Blotting

Cell or tissue were lysed or homogenized in a lysis buffer containing 50 mM Tris-HCl (pH 8.0), 150 mM NaCl, 1% NP-40, and 12 mM Na-deoxycholate supplemented with protease and phosphatase inhibitors cocktail. Nuclei and cytosol were obtained as previously described [26]. Protein concentration was determined by the Lowry protein assay. Proteins were separated by SDS-PAGE and transferred to nitrocellulose membranes that were probed with the following antibodies: anti-*n*NOS (Santa Cruz), anti-SOD1 (Santa Cruz), anti-LDH (Santa Cruz), anti-poly-ADP-ribose polymerase 1 (PARP1), anti-actin (Santa Cruz), anti-tubulin (Sigma), anti-GSNOR (Thermo Scientific), anti-DJ-1 (Santa Cruz), anti-AKT (Santa Cruz), anti-phospho-AKT (Santa Cruz), anti-Nrf2 (Santa Cruz), and anti-H2B (Santa Cruz). After immunostaining with appropriate secondary horseradish peroxidase-conjugated goat anti-rabbit or anti-mouse antibodies, bands were revealed using the Amersham ECL detection system.

### 2.7. Detection of Oxidative Stress

#### 2.7.1. ROS Evaluation

30 minutes before the end of the experimental time, cells were incubated with 50 *μ*M H_2_-DCF at 37°C, washed, and resuspended in ice-cold PBS. The fluorescence intensities of DCF, formed by the reaction of H_2_-DCF with ROS, were analyzed cytofluorometrically by recording FL-1 fluorescence.

#### 2.7.2. Carbonylation

Carbonylated proteins were detected using the Oxyblot Kit (Intergen, Purchase, NY) after reaction with 2,4-dinitrophenylhydrazine (DNP) for 15 minutes at 25°C. Samples were then resolved by 10% SDS polyacrylamide gel electrophoresis and DNP-derivatized proteins identified by immunoblot using an anti-DNP antibody.

### 2.8. Evaluation of GSNOR Activity

GSNOR activity was evaluated spectrophotometrically in clarified cell or spinal cord lysates at 340 nm as previously described [20, 24].

### 2.9. Detection of* S*-Nitrosylated Proteins (PSNOs) and Pull-Down

*S*-Nitrosylation assays were performed as previously described [27]. In particular, cells or tissues were lysed or homogenized in HEN buffer (25 mM HEPES, 50 mM NaCl, 0.1 mM EDTA1% NP-40, protease inhibitors, pH 7.4). Free cysteine residues were blocked with* S*-methyl methanethiosulfonate (MMTS). More specifically, 0.5–2 mg of protein samples were diluted to 1.8 mL with HEN buffer (100 mM HEPES, 1 mM EDTA, 0.1 mM neocuproine, pH 8.0). Next, 0.2 mL of 25% SDS is added along with 20 *μ*L of 10% MMTS (to reach a final volume of 2 mL and final concentrations of 2.5% SDS and 0.1% MMTS) and incubated at 50°C in the dark for 15–20 min with frequent vortexes. Proteins were then precipitated upon incubation with acetone, resuspended in 0.24 mL HENS buffer (HEN buffer with 1% SDS), and transferred to a fresh tube containing biotin-HPDP (2.5 mg/mL) with or without sodium ascorbate (20 mM). After incubation with the HRP-conjugated streptavidin, biotinylated proteins were revealed using the Amersham ECL detection system. Ascorbate-free samples have been omitted for straightforward visualization and interpretation of data.

Once obtained, the pool of PSNOs was pulled down by incubation with streptavidin and proteins there included were revealed on SDS-PAGE upon incubation with specific primary antibody (e.g., DJ-1).

### 2.10. Fluorescence Microscopy

Cells were cultured on coverslips, fixed with 4% paraformaldehyde, and permeabilized. Polyclonal anti-Nrf2 and monoclonal Grp75 (Millipore) were used as primary antibodies and probed successively with the appropriate Alexa Fluor 568-conjugated secondary antibody. To visualize nuclei and the actin cytoskeleton, cells were incubated with the cell-permeable DNA-specific dye Hoechst 33342 (Calbiochem-Novabiochem) and fluorescein isothiocyanate- (FITC-) conjugated phalloidin (Invitrogen), respectively. Images were digitized with a Cool Snap video camera connected to a Nikon Eclipse TE200 fluorescence microscope.

### 2.11. Fluorescence Microscopy Detection of Δ*ψ*_*m*_

JC-1 (Life Technologies) was added to prewarmed cell medium at the final concentration of 5 *μ*g/mL in order to evaluate Δ*ψ*_*m*_. After incubation for 10 min at 37°C, cells were washed in PBS and analyzed using a EVOS Floid Cell Imaging Station.

### 2.12. Data Presentation

All experiments were done at least three different times unless otherwise indicated. The results are presented as means ± SD. Statistical evaluation was conducted by ANOVA, followed by Bonferroni's test. Comparisons were considered significant at *P* < 0.05.

## 3. Results

### 3.1. GSNOR Downregulation Protects SH-SY5Y Cells from MPP^+^-Induced Toxicity

**Figure 1:**
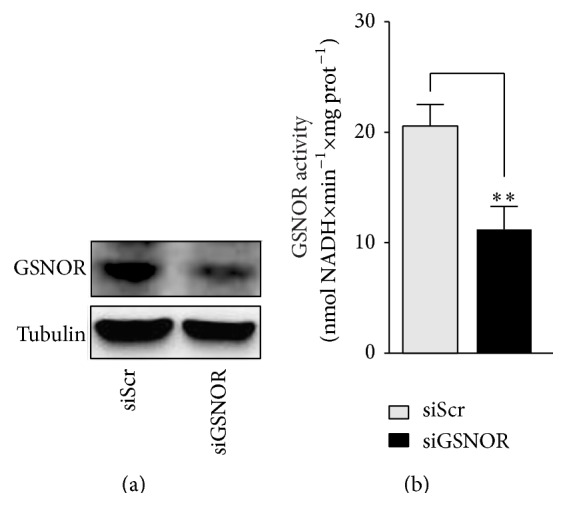
Characterization of siGSNOR cells. (a) Western blotting of GSNOR in total cell lysates of SH-SY5Y cells transiently transfected for 18 h with siRNA against GSNOR (siGSNOR) or control scramble siRNA (siScr). (b) Analyses of GSNOR activity in siGSNOR and siScr cells. Western blots shown are representative of at least *n* = 3 independent experiments that gave similar results. Tubulin was selected as loading control. Graphs shown represent the mean of data ± SD of *n* = 3 independent experiments, ^*∗∗*^ *P* < 0.01.

**Figure 2:**
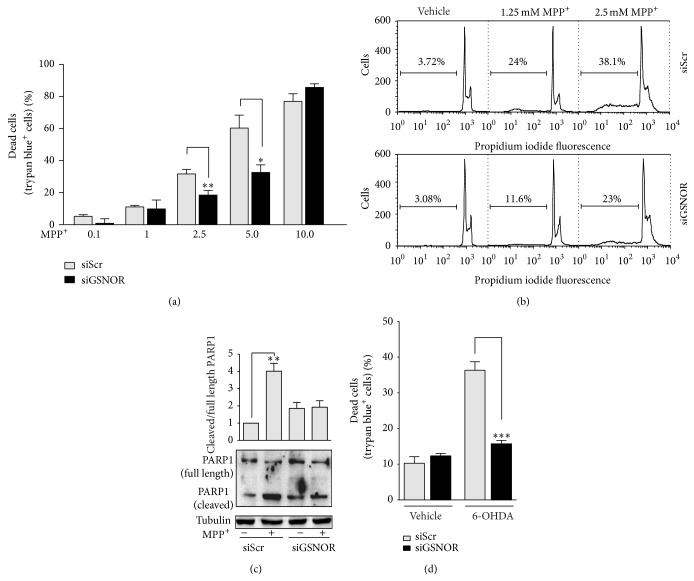
Cell death in siGSNOR cells. (a) Direct cell count upon Trypan blue staining of siScr and siGSNOR SH-SY5Y cells treated for 24 h with 0.1 to 10 mM MPP^+^. (b) Cytofluorimetric histogram of siScr and siGSNOR SH-SY5Y cells treated for 24 h with 1.25 or 2.5 mM MPP^+^ upon propidium iodide staining percentage of subG1 (apoptotic) population is shown. (c) Western blotting of PARP1 in total cell lysates of siScr and siGSNOR SH-SY5Y cells treated for 24 h with or without 2.5 mM MPP^+^. Densitometric analyses reporting the ratio cleaved: full length of PARP1 immunoreactive bands are shown on the top. (d) Direct cell count upon Trypan blue staining of siScr and siGSNOR SH-SY5Y cells treated for 24 h with 50 *μ*M 6OHDA. Western blots shown are representative of at least *n* = 3 independent experiments that gave similar results. Tubulin was selected as loading control. Graphs shown represent the mean of data ± SD of *n* = 3 independent experiments, ^*∗*^ *P* < 0.05, ^*∗∗*^ *P* < 0.01, and ^*∗∗∗*^ *P* < 0.001.

In order to analyze the effects of* S*-nitrosylation on the viability of dopaminergic cells challenged with PD toxins, we selected the SH-SY5Y cell line in which we transiently knocked down GSNOR (siGSNOR cells) (Figures 1(a) and 1(b)). We then subjected the cells to different doses of the mitochondrial toxin MPP^+^, used to recapitulate* in vitro* PD-like conditions, and we analyzed cell viability by Trypan blue staining. Dose response experiments indicted that, except for the highest dose employed (namely, 10 mM) where viability was completely compromised, siGSNOR cells were more resistant to MPP^+^-mediated toxicity (Figure 2(a)). Cytofluorometric analyses upon propridium iodide staining also demonstrated that the extent of Sub-G1 (apoptotic) population was lower in siGSNOR cells (Figure 2(b)) upon treatment with either 1.25 or 2.5 *μ*M MPP^+^. Coherently, Western blot analyses of poly-ADP ribose polymerase 1 (PARP1) cleavage indicated that siGSNOR cells were almost unaffected by treatment with 2.5 mM MPP^+^ if compared with the scrambled siRNA transfected controls (siScr) (Figure 2(c)). To confirm these results, we also treated the cells with 50 *μ*M 6-hydroxydopamine (6-OHDA), another toxin usually employed to induce PD-like cell death. As expected, results shown in Figure 2(d) confirmed that siGSNOR cells were more resistant to PD toxin mediated challenge.

### 3.2. GSNOR Downregulation Correlates with a General Decrease of Oxidative Stress

**Figure 3:**
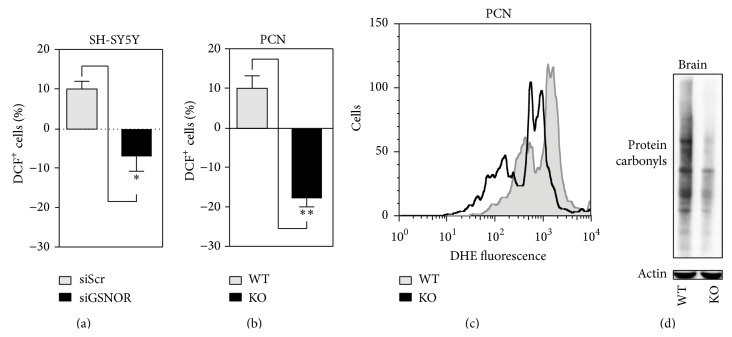
ROS production and protein carbonylation in GSNOR-downregulating systems. (a) Cytofluorimetric analyses of H_2_O_2_ in siScr and siGSNOR SH-SY5Y cells upon 2′,7′-dihydrodichlorofluorescein (DCF) staining. (b) Cytofluorimetric analyses of H_2_O_2_ and O_2_^•−^ in GSNOR-KO and WT PCN upon 2′,7′-dihydrodichlorofluorescein (DCF) or (c) dihydroethidine (DHE) staining. (d) Western blot analyses of protein carbonyls from mesencephalon obtained from GSNOR-KO and WT brains. Western blots shown are representative of at least *n* = 3 independent experiments that gave similar results. Actin was selected as loading control. Graphs shown represent the mean of data ± SD of *n* = 3 independent experiments, ^*∗*^ *P* < 0.05 and ^*∗∗*^ *P* < 0.01.

In order to figure out the molecular events responsible for siGSNOR cell resistance to PD toxins, we cytofluorometrically analyzed H_2_O_2_ produced in SH-SY5Y under basal conditions by means of incubations with 2′,7′-dihydrodichlorofluorescein (DCF).  Figure 3(a) indicates that H_2_O_2_ production was endogenously lower in siGSNOR than siScr cells. Consistently, primary cortical neurons (PCN) obtained from GSNOR-deficient (GSNOR-KO) mice showed an endogenous production of H_2_O_2_ significantly reduced with respect to the wild type (WT) counterparts (Figure 3(b)). Dihydroethidine (DHE) staining also showed that GSNOR-KO PCN exhibited low levels of O_2_^•−^ (Figure 3(c)), confirming that GSNOR depletion was associated with a general reduction of endogenous levels of ROS. These lines of evidence were in agreement with the observation that the extent of protein carbonylation measured in lysates from mesencephalon of brains obtained by GSNOR-KO mice was lower than the WT counterpart (Figure 3(d)), suggesting that GSNOR deficiency could induce an enhancement of the antioxidant defense.

### 3.3. GSNOR Downregulation Induces DJ-1 and Nrf2 Activation

**Figure 4:**
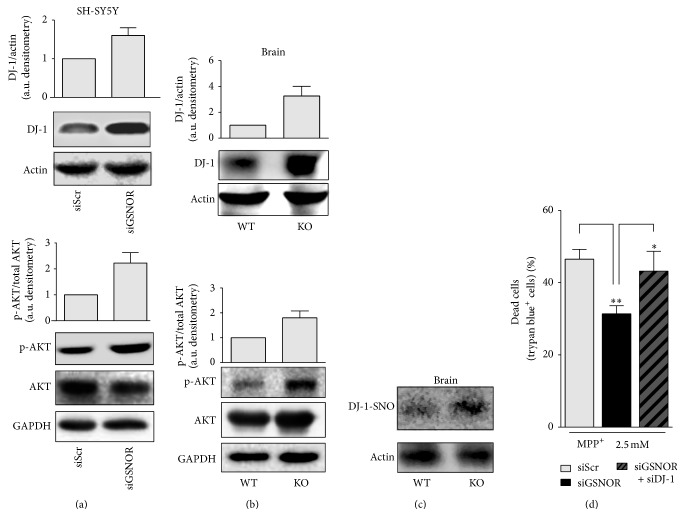
DJ-1 and AKT in siGSNOR cells and GSNOR-KO brains. Western blot analyses of DJ-1, as well as basal and phosphoactive AKT in total extracts obtained from (a) siScr and siGSNOR SH-SY5Y cells or (b) GSNOR-KO and WT brains. Densitometric analyses of DJ-1 and phospho-AKT are shown on the top of the corresponding Western blot normalized to actin and basal AKT, respectively. (c) Biotin switch assay followed by pull-down with streptavidin and revealed with anti-DJ-1 antibody, in total extracts obtained from GSNOR-KO and WT brains. Western blot analysis indicates that DJ-1 was* S*-nitrosylated (present in the pull-down). Western blots shown are representative of at least *n* = 3 independent experiments that gave similar results. Actin or GAPDH were selected as loading controls. (d) Direct cell count upon Trypan blue staining of siScr and siGSNOR SH-SY5Y cells transfected or not with siRNA against DJ-1 (siDJ-1) and treated for 24 h with 2.5 mM MPP^+^. Graphs shown represent the mean of data ± SD of *n* = 3 independent experiments, ^*∗*^ *P* < 0.05 and ^*∗∗*^ *P* < 0.01.

In the search for the molecular determinants underlying the decrease of oxidative markers observed upon GSNOR downregulation or complete ablation, we focused on DJ-1, whose increased expression and* S*-nitrosylation have been reported playing a pivotal role in neuroprotection [9, 28, 29]. Results shown in Figure 4(a) indicate that DJ-1 was upregulated in siGSNOR SH-SY5Y cells and that this correlated with an increase in the phosphoactive form of AKT. Interestingly, total brain lysates obtained from WT and GSNOR-KO mouse brains displayed the same trend (Figure 4(b)), which was also associated with an increase of the* S*-nitrosylated form of DJ-1 (Figure 4(c)), suggesting that DJ-1 was upregulated and, reasonably, activated by* S*-nitrosylation upon GSNOR downregulation. Coherently, transfection with siRNA against DJ-1 resulted in a significant increase of MPP^+^-mediated toxicity in siGSNOR cells, while it seemed to be completely ineffective in siScr SH-SY5Y (Figure 4(d)).

**Figure 5:**
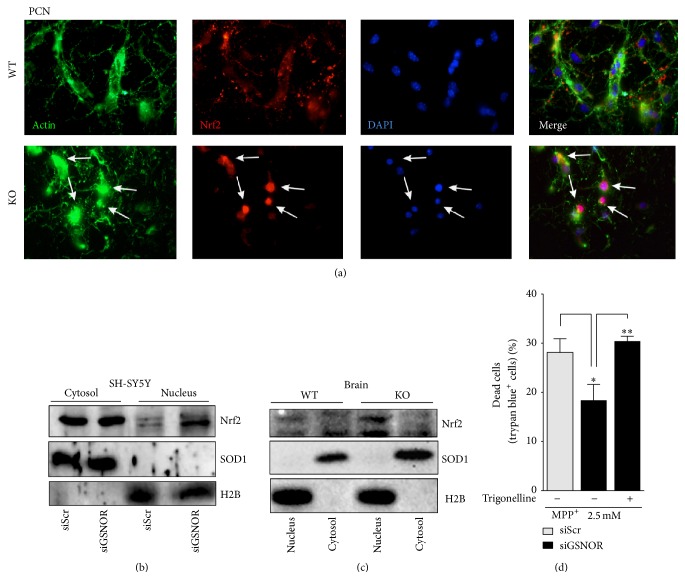
Nuclear localization of Nrf2 in GSNOR-downregulating systems. (a) Fluorescence microscopy analyses of WT and GSNOR-KO PCN upon staining with anti-Nrf2 antibody (red), phalloidin (green), and Hoechst 33342 (blue), with the last two probes used to selectively highlight actin (cytoskeleton) and nuclei, respectively. Western blot analyses of Nrf2 in nuclear and cytosolic fractions obtained from (b) siScr and siGSNOR SH-SY5Y cells or (c) GSNOR-KO and WT brains. Western blots shown are representative of at least *n* = 3 independent experiments that gave similar results. SOD1 and histone 2B (H2B) were selected as loading and purity controls of cytosol and nuclei, respectively. (d) Direct cell count upon Trypan blue staining of siScr and siGSNOR SH-SY5Y cells treated for 24 h with 2.5 mM MPP^+^ in the presence or absence of 1 mM of the Nrf2 pharmacological inhibitor trigonelline. Graphs shown represent the mean of data ± SD of *n* = 3 independent experiments, ^*∗*^ *P* < 0.05 and ^*∗∗*^ *P* < 0.01.

We previously demonstrated that GSNOR-KO skeletal muscle showed an induction of Nrf2 [23]. Moreover data from the literature show that DJ-1 is an upstream modulator of the Nrf2-mediated antioxidant response [10, 11]. Therefore, we focused on this transcription factor. Namely, we analyzed its nuclear* versus* cytosolic localization, which is commonly accepted as predictive sign of Nrf2 activation. Immunofluorescence analyses of PCN indicated that Nrf2 localization significantly changed from being exclusively cytosolic and axonal (WT) into predominantly nuclear (GSNOR-KO) (Figure 5(a)). Western blot analyses performed in cytosolic and nuclear fractions of both siGSNOR SH-SY5Y and GSNOR-KO brains indicated that nuclear levels of Nrf2 were higher if compared with siScr and WT controls, respectively (Figures 5(b) and 5(c)), confirming previously obtained results and reinforcing the hypothesis that Nrf2 was activated upon GSNOR downregulation. In agreement with these observations, we inhibited Nrf2 with 1 *μ*M of its pharmacological inhibitor trigonelline and analyzed cell death.  Figure 5(d) shows that preincubation with trigonelline significantly exacerbated MPP^+^-induced toxicity in siGSNOR cells, arguing for a role of Nrf2 in cell protection against MPP^+^ in conditions of nitrosative stress.

### 3.4. GSNOR Decrease Sensitizes G93A-Expressing Models of ALS to Nitrosative Stress

**Figure 6:**
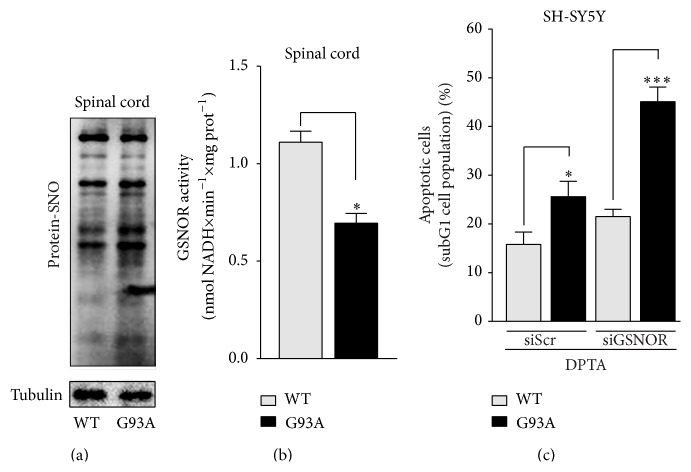
Characterization and sensitivity of G93A models to nitrosative stress. Biotin switch assay of total protein-SNOs (a) and analysis of GSNOR activity (b) performed on spinal cord lysates of WT and G93A-SOD1 expressing mice (G93A). (c) Cytofluorimetric analyses of apoptosis upon propidium iodide staining performed on parental SH-SY5Y cells (WT) and G93A-SOD1 mutants (G93A) transfected or not with siRNA against GSNOR (siGSNOR) and treated for 24 h with 400 *μ*M DPTA. Western blots shown are representative of at least *n* = 3 independent experiments that gave similar results. Tubulin was selected as loading control. Graphs shown represent the mean of data ± SD of *n* = 3 independent experiments, ^*∗*^ *P* < 0.05 and ^*∗∗∗*^ *P* < 0.001; n.s.: not significant.

Next, we wondered to extend our analyses and figure out any role of GSNOR in neurodegenerative disturbances where NO-mediated toxicity has been frequently reported being a factor affecting neuronal viability. In particular, we focused on ALS whose progression and severity have been tightly correlated with NO overproduction and nitrosative stress. In order to verify whether excessive* S*-nitrosylation occurred in ALS, we initially performed biotin switch assays of* S*-nitrosylated proteins (PSNOs) in spinal cord of mice overexpressing the fALS associated G93A mutant form of SOD1 that we selected as* in vivo* elective model of ALS; Figure 6(a) shows that PSNOs levels were significantly increased in symptomatic G93A mice (135-day-old), confirming the hypothesis that ALS was accompanied by aberrant* S*-nitrosylation. Interestingly, spectrophotometric analyses indicated that this condition was associated with a marked decrement of GSNOR activity (Figure 6(b)), suggesting that GSNOR decrease could concur to nitrosative stress and motor neuron death observed in ALS.

Coherently, we and others had already reported that SOD1-G93A overexpression rendered SH-SY5Y cells (hereafter named G93A cells) sensitive to nitrosative stress [30], and during our experiments we observed that 24 h treatments with 400 *μ*M of the NO-donor DPTA induced higher extent of apoptotic cells in G93A cells if compared with parental SH-SY5Y cells (Figure 6(c)). Interestingly, this condition was exacerbated by siRNA-induced GSNOR downregulation (Figure 6(c)), adding in such a way a further evidence that GSNOR could play a role in ALS, namely, in protecting ALS motor neurons against nitrosative stress.

### 3.5. GSNOR Upregulation Induces Resistance to Nitrosative Stress in G93A Cells

**Figure 7:**
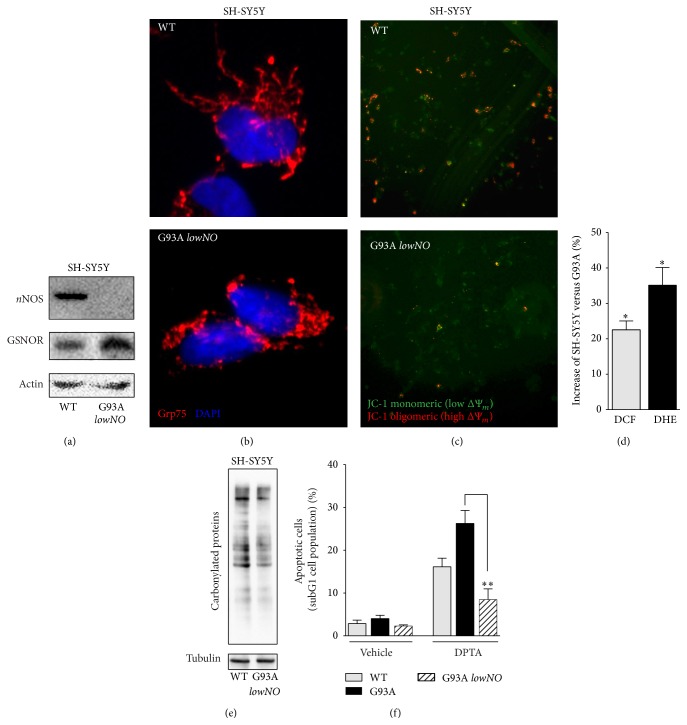
Characterization of the* lowNO* G93A resistant clone. (a) Western blotting of* n*NOS and GSNOR in total cell lysates of parental SH-SY5Y (WT) and the resistant* lowNO* G93A clone. (b) Fluorescence microscopy analyses of parental SH-SY5Y (WT) and the* lowNO* G93A clone. Anti-Grp75 (red) and Hoechst 33342 were used to visualize mitochondria and nuclei, respectively. (c) Fluorescence microscopy analyses of mitochondrial transmembrane potential (Δ*ψ*_*m*_) of WT and the* lowNO* G93A clone by JC-1. Red fluorescence, high Δ*ψ*_*m*_ and green fluorescence, low Δ*ψ*_*m*_. (d) Cytofluorimetric analyses of H_2_O_2_ and O_2_^•−^ in SH-SY5Y (WT) and the* lowNO* G93A clone upon 2′,7′-dihydrodichlorofluorescein (DCF) or (c) dihydroethidine (DHE) staining. (e) Western blot analyses of protein carbonyls from total cell lysates obtained from WT and the* lowNO* G93A clone. (f) Cytofluorimetric analyses of apoptosis upon propidium iodide staining performed on parental SH-SY5Y cells (WT), sensitive G93A, and the* lowNO* G93A clone treated for 24 h with 400 *μ*M DPTA. Western blots shown are representative of at least *n* = 3 independent experiments that gave similar results. Actin or tubulin was selected as loading controls. Graphs shown represent the mean of data ± SD of *n* = 3 independent experiments, ^*∗*^ *P* < 0.05 with respect to trigonelline untreated cells.

We then selected a clone of G93A cells that significantly upregulated GSNOR and downregulated* n*NOS (clone hereafter named as* lowNO*) (Figure 7(a)). This clone still maintained the typical phenotype of fALS mutant SOD1, such as mitochondrial fragmentation (Figure 7(b)) and depolarization (Figure 7(c)), together with an endogenous production of ROS (Figure 7(d)). However, the extent of protein carbonylation decreased in this clone (Figure 7(e)), reinforcing the idea that NO directly contributes to carbonylation.

In agreement with high GSNOR levels, we found out that the* lowNO* clone was highly resistant to 24 h treatment with DPTA (Figure 7(f)), indicating that GSNOR induction was protective against nitrosative challenge in* in vitro* fALS models.

### 3.6. Nrf2 Contributes to Antinitrosative Response

**Figure 8:**
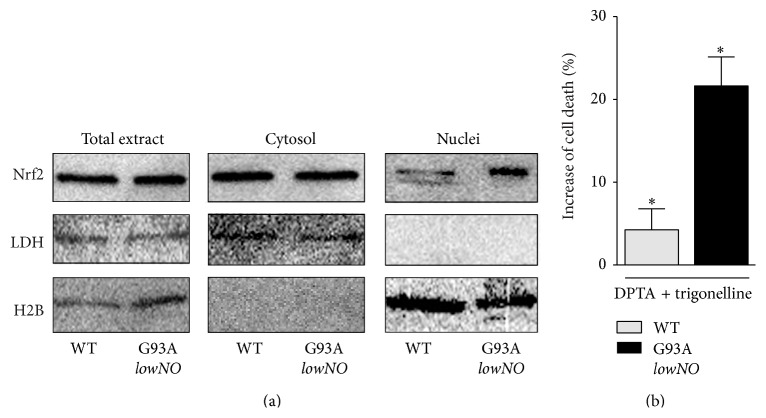
Role of Nrf2 in the* lowNO* G93A resistant clone. (a) Western blot analyses of Nrf2 in nuclear and cytosolic fractions obtained from parental SH-SY5Y (WT) and the* lowNO* G93A clone. (b) Cytofluorimetric analyses of apoptosis upon propidium iodide staining performed on parental SH-SY5Y cells (WT) and the* lowNO* G93A clone treated for 24 with DPTA in the presence or absence of the Nrf2 inhibitor trigonelline (0.5 *μ*M). Western blots shown are representative of at least *n* = 3 independent experiments that gave similar results. Lactate dehydrogenase (LDH) and histone H2B were selected as loading and purity controls. Graphs shown represent the mean of data ± SD of *n* = 3 independent experiments.

In order to characterize the GSNOR-based preconditioning response underlying* lowNO* clone resistance to nitrosative stress, we performed localization assays of Nrf2 by Western blot.  Figure 8(a) shows that Nrf2 preferentially distributed in the nucleus of* lowNO* clone, arguing for Nrf2 being induced in these cells. Consistently, incubation of* lowNO* clone with trigonelline increased susceptibility to DPTA (Figure 8(b)), allowing the hypothesis that Nrf2 activation and GSNOR upregulation, both found in* lowNO* clone, were functionally correlated in mediating resistance of G93A cells to nitrosative stress.

## 4. Discussion

In this work we have analyzed and characterized the role of GSNOR and* S*-nitrosylation in modulating phenotype severity of* in vitro* PD and fALS models. Despite the fact that GSNOR is the only alcohol dehydrogenase highly expressed in the central nervous system of mouse, rat, and humans [24], to the best of our knowledge this is the first evidence arguing for GSNOR being a crucial molecular player of neuronal homeostasis. Interestingly, here we have provided evidence that knocking down GSNOR expression results in a significant protection against PD toxins, such as MPP^+^ and 6OHDA. We recently demonstrated that muscles from GSNOR-KO mice show a clear fragmentation and depolarization of the mitochondrial network, which are associated with an endogenous activation of Nrf2-mediated antioxidant response [23]. It is therefore plausible that, under these conditions, mitochondria are less affected by MPP^+^ and highly protected against any further production of ROS, for example, that induced by 6OHDA. From this perspective, GSNOR downregulation represents a molecular event responsible for neuronal cell adaptation against oxidative stress and a possible tool to be exploited in order to counteract PD onset and progression.

However, GSNOR downregulation should sensitize cells to nitrosative stress. This is a crucial issue if we take into account neurodegenerative diseases in which NO-mediated toxicity highly contributes to the loss of specific neuronal populations, such as motor neurons in ALS. In particular, a direct role of chronic NO-based inflammatory response activated by astrocytes and microglia has been reported in ALS as detrimental event concurring to motor neuron cell death [4]. In agreement, we observed that* in vitro* models of fALS, namely, the SOD1-G93A SH-SY5Y cells, are highly susceptible to the toxicity of the NO-releasing drug DPTA, which is in line with previously published data obtained with other NO donors [30]. In these conditions, ectopic downregulation of GSNOR expression by siRNA exacerbates NO-mediated cell death, confirming our hypothesis of a protective role of GSNOR in cells susceptible to nitrosative stress. Remarkably, in our studies we found out that G93A-SOD1 expressing mice show an increase of spinal cord PSNOs in association with a decrease of GSNOR activity. This finding suggests for the first time that GSNOR is downregulated in ALS and argues for GSNOR decrease being a new marker of ALS onset and, mainly, progression. This hypothesis is strengthened by the results obtained in the GSNOR-overexpressing* lowNO* G93A clone which shows resistance to DPTA, thereby indicating that* S*-nitrosylation is a posttranslational modification strongly implicated in driving motor neuron death response to nitrosative stress in ALS.

Interestingly, our data support the hypothesis that Nrf2 could play a pivotal role also in these conditions. In particular, we have found out that cell adaptation induced by* lowNO* G93A clone to survive to nitrosative stress conditions (namely, GSNOR induction combined with* n*NOS downregulation) is associated, at least in part, with the induction of Nrf2, as trigonelline significantly increases* lowNO* G93A clone sensitivity to NO.

Altogether, these results argue for Nrf2 being tightly related to GSNOR expression. In particular, while Nrf2 is activated in response to (downstream of) GSNOR depletion to protect against PD toxins-induced cell death, it could also induce (be upstream of) GSNOR transcription to preserve survival in* in vitro* models of fALS in conditions of nitrosative stress. A positive feedback loop between Nrf2 and GSNOR could be therefore hypothesized to occur in order to sustain cell viability in conditions of nitrooxidative stress. However, if GSNOR is a direct target of Nrf2 still waits to be demonstrated.
